# Safety of Inactivated Influenza Vaccine in Patients with Egg Allergy in Kurdistan Province, Iran

**Published:** 2019-04

**Authors:** Sima TOZANDEHJANI, Rasoul NASIRI KALMARZI, Mazaher KHODABANDEHLOO, Hajar KASHEFI

**Affiliations:** 1. Department of Biological Sciences, Faculty of Science, University of Kurdistan, Sanandaj, Iran; 2. Lung Diseases and Allergy Research Center, Kurdistan University of Medical Sciences, Sanandaj, Iran; 3. Cellular & Molecular Research Center, Kurdistan University of Medical Sciences, Sanandaj, Iran; 4. Social Determinants of Health Research Center, Kurdistan University of Medical Sciences, Sanandaj, Iran

**Keywords:** Safety, Egg allergy, Inactivated influenza vaccine, Side effects

## Abstract

**Background:**

Most influenza vaccines are grown in embryonated eggs and residual egg proteins can cause allergic reactions in patients with egg allergy. The aim of the present study was to determine the safety of inactivated influenza vaccine in patients with egg allergy in Kurdistan Province, Iran.

**Methods:**

This case-control study was done on 876 patients referred to Kurdistan Asthma and Allergy Clinic, Sanandaj, Iran; 635 patients with egg allergy (cases) and 241 patients without egg allergy (controls) from 2012 to 2016. All of the patients were injected seasonal influenza vaccine. Side effects including anaphylactic shock, local reaction, vomiting, coughing, sneezing, wheezing, low blood pressure, redness and itching in the eyes, abdominal pain, dyspnea, oral/facial angioedema, swollen and itching of throat were checked by an allergist within 30 min after vaccination, and followed up to 24 h. Demographic and vaccination data were entered into the SPSS software and analyzed.

**Results:**

Out of 876 patients, 460 (52.5%) were male. Patients’ ages ranged from 6 months to 80 yr (mean 13.38 ± 15.22 SD). Overall, 63 patients with egg allergy had local reactions to vaccine. Difference of local reactions between case and control groups was statistically significant (*P*=0.001). No anaphylactic reactions were seen after vaccination.

**Conclusion:**

Although the risk of anaphylactic reactions to influenza vaccine in patients with egg allergy was rare, the vaccine should be administered by an allergist with precaution. The results of present study can be a confirmation of the existing evidences to prevent acute complications to influenza vaccine.

## Introduction

Hypersensitivity reactions are exaggerated immune responses to previously encountered antigens. Such reactions are divided into four types; type I or IgE-mediated hypersensitivity most often called allergy. In this type of hypersensitivity, IgE antibodies are produced against antigens (allergens). IgE antibodies bound to mast cells, and with subsequent exposure to the allergen, the mast cell-bound IgE triggers cell degranulation, and release of immunologic mediators such as histamine, leading to clinical signs of allergy. The most serious form of IgE-mediated hypersensitivity is called anaphylaxis, a serious form of systemic allergic reaction, that is rapid in onset, occurring within seconds to minutes, and involves multiple organs typically dermatologic/mucosal, cardiovascular, respiratory, and gastrointestinal; and may cause death (1, 2).

Egg allergy is one of the most common food allergies in children, at least 2% of preschool children (3). Mild symptoms of egg allergy are seen as hives only. Severe symptoms (e.g., anaphylaxis) involve cardiovascular (e.g., hypotension), respiratory (e.g., wheezing, dyspnea), and gastrointestinal (e.g., nausea, vomiting), generally require epinephrine or emergency treatment. Egg allergy may correlate with respiratory symptoms (asthma and/or rhinitis) (2). Egg allergy can be confirmed by a consistent history of adverse reactions to ingestion of egg and egg-containing foods, skin and/or blood testing for IgE produced against egg proteins (4, 5).

Influenza viruses are the most abundant and the most important etiology of human respiratory infections (6). In infants, the elderly, and people with chronic diseases such as lung, heart, kidney, cancer, and organ transplantation, influenza is associated with more death. In addition, unvaccination may cause morbidity and mortality in children and high-risk groups such as elderly patients (1, 7).

Most influenza vaccines are grown in embryonated eggs and inactivated (8). For example, monovalent influenza type A vaccine, containing only strain H1N1 (9, 10), trivalent inactivated influenza vaccine (IIV3), usually contains two strains of influenza virus type A, and one strain of influenza virus type B (11). Quadrivalent inactivated influenza vaccine (IIV4) contains four strains of influenza viruses, two strains A and two strains B. All administrated by injection. Finally, a live vaccine termed quadrivalent live attenuated influenza vaccine (LAIV4), administered intranasally (1).

Until just recent years, the administration of influenza vaccine to patients with egg allergy was contraindicated, because injection of influenza vaccines that contained remnant of egg proteins to a person with egg allergy, that produces IgE antibodies against egg proteins, could cause anaphylaxis (12). Some of the developed methods, that made possible to receive safely inactivated influenza vaccine in patients with egg allergy, including gradual vaccination, skin testing of the vaccine, and use of a vaccine with low-level oval-bumin (8).

Recently, in order to get rid of residual egg proteins from influenza vaccines, two new non-egg-based influenza vaccines have been developed. One involves influenza viruses grown in mammalian cell culture, available as trivalent cell culture inactivated influenza vaccine (ccIIV3). The other vaccine involves influenza virus recombinant hemagglutinin protein expressed in an insect cell line and is available as trivalent recombinant influenza vaccine (RIV3) (1, 12). However, in countries where egg-free influenza vaccines do not prevail, patients with egg allergy could be given the IIV3 vaccine with precaution.

IIV3 was safe for patients with egg allergy, and no need any specific precautions to receive influenza vaccine (2, 7, 8). However, numbers of such studies are limited. In addition, there was no published information on the safety of IIV3 in patients with egg allergy in Iran.

Therefore, the aim of present study was to determine the possible side effects of seasonal injectable trivalent inactivated influenza vaccine (IIV3) in patients with egg allergy in Kurdistan Province, west of Iran.

## Methods

### Study population

Kurdistan Province located in the west of Iran. According to the last census conducted in 2011, Kurdistan Province had a total population of 1493645. The male/female ratio of the population was 1.1 and most of populations are Kurd.

We were able to get information from 876 patients referred to the Kurdistan asthma and allergy clinic for routine medical services from 2012 to 2016. Out of 876 study population, 635 patients had allergy to egg, confirmed with a consistent medical history of adverse reactions to ingestion of egg and egg-containing foods and/or positive skin test and 241 patients without egg allergy. 56 patients suffered from asthma, 369 from rhinitis, and 76 had a food allergy, except egg.

In this case-control study, 635 patients with egg allergy (cases) and 241 patients without egg allergy (controls) were administered seasonal trivalent inactivated influenza vaccine (IIV3), subunit vaccine (INFULVAC, Abbott Biologicals, Netherlands). The vaccine was injected (0.5 ml for adults and children above 36 months; and 0.25 ml for 6–35 months children) under medical supervision.

Probable side effects to the vaccine including anaphylactic shock, local reaction (redness of skin), systemic reaction (general skin reaction or tachycardia), vomiting, coughing, sneezing, stridor, wheezing, low blood pressure, redness and itching in the eyes, dyspnea, oral/facial angioedema, swollen/itching of throat, and abdominal pain were checked by an allergist within 30 min after vaccination, and followed up to 24 h by telephone. Patients with a history of wheeze or asthma underwent further follow up.

Demographic information including age, gender, and other patients’ clinical conditions were collected.

### Statistical analysis

Demographic and vaccination information were entered into the SPSS software (ver.19 (Chicago, IL, USA) and were analyzed by the chi-square statistical test. In all steps *P*-values, less than 0.05 were considered statistically significant.

### Ethical considerations

Patients voluntarily referred to the Kurdistan Asthma and Allergy Clinic for routine medical services. We get patients’ information from the clinic archive. The patients’ names and their information were held confidential. This study was approved at Kurdistan University of Medical Sciences, Sanandaj, Iran (proposal and ethics code: IR.MUK.REC.1395/362).

## Results

Out of 876 patients, 460 (52.5%) were male and 416 (47.5%) were female. The age range of the patients was 6 months to 80 yr (mean 13.38 ± 15.22 Std. deviation). No anaphylactic reactions or shocks were seen after administration of seasonal injectible inactivated influenza vaccine in both groups. However, there were some minor reactions to the vaccine. For example, 63 out of 635 patients with egg allergy (cases) had local reactions (redness of skin) to the vaccine. However, there were no local reactions to the vaccine in patients without egg allergy (controls). Difference of the local reactions between case and control groups was statistically significant (*P*=0.001). Other minor reactions to influenza vaccine are presented in Table 1.

**Table 1:** The comparison of side effects to seasonal injectible inactivated influenza vaccine in patients with egg allergy (cases) and patients without egg allergy (controls), according to chi-square statistical test

***Variable***	***Result***	***Cases Frequency (percent)***	***Controls Frequency (percent)***	***Pearson chi-square statistics***	***P-value***
Local reaction (redness of skin)	Negative	572 (70.4)	241 (29.6)	26.76	0.001
	Positive	63 (100)	0 (0)		
Systemic reaction (General skin reaction or tachycardia)	Negative	631 (72.4)	241 (27.6)	1.53	0.341
	Positive	4 (100)	0 (0)		
Oral/facial angioedema	Negative	633 (72.4)	241 (27.6)	0.761	0.252
	Positive	2 (100)	0 (0%)		
Itching of throat	Negative	626 (72.5)	238 (27.5)	0.038	0.570
	Positive	9 (75)	3 (25)		
Swollen of throat	Negative	633 (72.5)	240 (27.5)	0.051	0.620
	Positive	2 (66.7)	1 (33.3)		
Stridor	Negative	631 (72.4)	241 (27.6)	1.525	0.580
	Positive	4 (100)	0 (0)		
Coughing and sneezing	Negative	631 (72.4)	241 (27.6	1.525	0.580
	Positive	4 (100)	0 (0%)		
Dyspnea	Negative	634 (72.5)	241 (27.5)	0.380	0.725
	Positive	1 (100)	0 (0)		
Wheezing	Negative	634 (72.5)	241 (27.5)	0.380	0.725
	Positive	1 (100)	0 (0)		
Low blood pressure	Negative	634 (72.5)	241 (27.5)	0.380	0.725
	Positive	1 (100)	0 (0)		
Abdominal pain	Negative	633 (72.4)	241 (27.6)	0.761	0.525
	Positive	2 (100)	0 (0)		
Redness and itching in the eyes	Negative	622 (72.4)	237 (27.6)	0.138	0.478
	Positive	13 (76.5)	4 (23.5)		

In patients with egg allergy 4 had systemic response to vaccination, 2 oral/facial angioedema, 9 itching of throat, 2 swelling of the throat, 4 stridor, 4 cough and sneeze, 1 dyspnea, 1 wheezing, 1 hypotension, 2 abdominal pain, and 13 redness and itching in the eyes, respectively. Difference of these minor reactions to inactivated influenza vaccine between case and control groups was not statistically significant (Table 1).

## Discussion

At present study, 635 patients with egg allergy (cases), and 241 patients without egg allergy (controls) were administered seasonal trivalent inactivated influenza vaccine (IIV3) in Kurdistan Asthma and Allergy Clinic in influenza seasons. No anaphylactic reactions were seen after vaccination. However, there were some minor reactions to vaccine. For examples, 63 patients with egg allergy had local reactions to vaccine. Difference of local reactions between case and control groups was statistically significant.

There are clear data regarding the safety of egg-protein containing influenza vaccines to patients with egg allergy. Review of 28 previous studies collectively showed that administration of egg-containing influenza vaccines to the patients with history of anaphylactic reactions to the ingestion of egg had no significant reactions (1, 12).

In many such studies, either prick or intradermal skin tests were done with the influenza vaccine before vaccination. The tests did not predict allergic reactions, and such testing was unnecessary. In addition, in many of the studies the vaccines were administered in divided doses (often 10% of dose followed up 30 min then the remaining 90%) (1, 12).

Because the full dose of the vaccine was tolerated, many of the authors concluded that dividing the dose was also unnecessary. In studies that included control subjects without egg allergy, the rate of minor reactions to vaccine was almost the same as in the recipients with egg allergy. Therefore, recipients scarcely developed anaphylactic reactions after administration of influenza vaccines in both egg allergic and non-allergic groups (1, 12).

Allergic/anaphylaxis reactions can occur to all types of vaccines in response to various components of vaccines, although, such reactions are rare. In over 25.1 million doses of various vaccines administered to children and adults, 33 cases of severe allergic reactions including anaphylaxis occurred (1.31 per 1 million vaccine doses). Among more than 7.4 million doses of IIV3 vaccine given, there were 10 cases of anaphylaxis (1.35 per 1 million doses) (5, 13). The Vaccine Adverse Event Reporting System (VAERS) has reported that egg-free recombinant influenza vaccine also caused rare allergic/anaphylaxis reactions among patients with egg allergy and patients allergic to IIV; thus reaction to vaccine is not inevitably related to remnant of egg proteins (14). Therefore, it is necessary to determine exactly other constituents of vaccine that cause anaphylaxis reactions and detect IgE response to vaccine constituents in recipients.

Ovalbumin as a marker for the egg protein in influenza vaccines may vary according to seasons, manufacturers, and vaccine lots (2). Nowadays all manufacturers of influenza vaccine indicate that their vaccine contains less than 1 μg of ovalbumin per dose. Although the allergic reaction to egg protein in vaccine varies from egg protein in the food, so far, the minimal dose of egg protein that can cause an allergic reaction has been 130 μg, and the amount of egg protein that cause no reactions in 99% of patients with egg allergy has been 30 μg. In this way, even in children with egg-allergy, the amount of egg protein in the influenza vaccines is not sufficient to stimulate allergic reactions (1). The median ovalbumin content of a seasonal and H1N1 influenza vaccines were 350 and 21 ng/ml, respectively (15). We did not detect egg protein (ovalbumin) content of administered influenza vaccine.

A cohort study using live attenuated influenza vaccine (LAIV), which contains less egg protein than IIV3, had no systemic reaction in the two hours after vaccination. Urticarial/angioedema was seen 30 to 120 min after LAIV administration in two recipients, but after weeks they received a second dose of LAIV without any reaction. Possible allergic reactions (local urticaria, nasal and oropharyngeal symptoms) were reported in nine patients. Lower respiratory tract symptoms within 72 h were reported in 62 patients (16). Therefore, the rate of reactions to vaccine is not related to amount of egg protein in the vaccine. We also had some similar minor reactions in our vaccine recipients. Maybe other constituents of vaccines cause reactions.

People with egg allergy are not at more risk to allergic reactions than general people, and it is not necessary for prohibition of inactivated influenza vaccine (8). Patients with egg allergy could be safely vaccinated with monovalent influenza A (H1N1) vaccine, although, those patients had anaphylactic reactions to egg (9). However, results of our study are compatible with previous studies.

We could not determine the concentration of egg proteins in the vaccines; in addition, we could not determine probable IgE antibodies to egg proteins or to another constituent of vaccines in our population. Among the strengths of this case-control study are the large sample size, clinical profile of patients with egg allergy and patients with asthma or rhinitis in our study population. Finally, administration of IIV3 in patients with egg allergy, and asthma or rhinitis seems to be safe.

## Conclusion

Although the risk of anaphylactic reactions to trivalent inactivated influenza vaccine in patients with egg allergy was rare, the vaccine should be administered by an allergist with precaution. The results of present study can be a confirmation of the existing evidences in order to prevent acute complications to influenza vaccine.

## Ethical considerations

Ethical issues (Including plagiarism, informed consent, misconduct, data fabrication and/or falsification, double publication and/or submission, redundancy, etc.) have been completely observed by the authors.
